# Active-Optical Sensors Using Red NDVI Compared to Red Edge NDVI for Prediction of Corn Grain Yield in North Dakota, U.S.A.

**DOI:** 10.3390/s151127832

**Published:** 2015-11-02

**Authors:** Lakesh K. Sharma, Honggang Bu, Anne Denton, David W. Franzen

**Affiliations:** 1 Cooperative Extension Service, University of Maine, 57 Houlton Road, Presque Isle, ME 04769, USA; E-Mail: lakesh.sharma@maine.edu; 2 Department of Soil Science, School of Natural Resource Sciences, North Dakota State University, Dept. 7180, PO Box 6050, Fargo, ND 58108, USA; E-Mail: honggang.bu@ndsu.edu; 3 Department of Computer Science, North Dakota State University, Dept. 2740, PO Box 6050, Fargo, ND 58108, USA; E-Mail: anne.denton@ndsu.edu

**Keywords:** corn, ground-based active-optical sensors, nitrogen, soil

## Abstract

Active-optical sensor readings from an N non-limiting area standard established within a farm field are used to predict yield in the standard. Lower yield predictions from sensor readings obtained from other parts of the field outside of the N non-limiting standard area indicate a need for supplemental N. Active-optical sensor algorithms for predicting corn (*Zea mays*, L.) yield to direct in-season nitrogen (N) fertilization in corn utilize red NDVI (normalized differential vegetative index). Use of red edge NDVI might improve corn yield prediction at later growth stages when corn leaves cover the inter-row space resulting in “saturation” of red NDVI readings. The purpose of this study was to determine whether the use of red edge NDVI in two active-optical sensors (GreenSeeker™ and Holland Scientific Crop Circle™) improved corn yield prediction. Nitrogen rate experiments were established at 15 sites in North Dakota (ND). Sensor readings were conducted at V6 and V12 corn. Red NDVI and red edge NDVI were similar in the relationship of readings with yield at V6. At V12, the red edge NDVI was superior to the red NDVI in most comparisons, indicating that it would be most useful in developing late-season N application algorithms.

## 1. Introduction

Numerous methods have been used to manage N application to crops, including zone soil sampling [1], soil and plant analysis [2], use of nitrogen credit from the influence of previous crops [3], tissue analysis [4], fertilizer application timing and placement [5,6,7,8], leaf area index [9,10,11,12,13], and the use of spectral sensors [2,14,15,16,17,18,19,20,21]. Normalized Differential Vegetative Index (NDVI) has been used for decades as a predictor of vegetative biomass [22].

The use of active-optical sensors to direct in-season N application is being used in wheat (*Triticum aestivum*, L.) and corn (*Zea mays*, L.) growing areas of the USA. The algorithms developed to direct in-season N application use one of two methods. One method is to establish an N non-limiting area within the field at the time of preplant N application [20] or an N-rate “ramp” consisting of a continuous series of increasing N rates with the highest N rate designed to be non-limiting to the crop [23]. The second method is to use the variability in greenness already in the field by using the greenest area within the field as the N non-limiting standard [24]. Once an NDVI measurement is performed on the N non-limiting standard, by either method, the result is that the greatest yield possible for the variety within the soil where the N non-limiting area is located is predicted. Sensor measurements within the field less than the standard sensor measurement result in lower yield predictions. The yield difference is used to calculate the N required to increase yield from its predicted value if no additional N was added to a yield predicted in the standard area.

Most of the algorithms developed for active-optical sensor use for in-season N fertilization utilize red NDVI. It is important to differentiate NDVI and specify red NDVI, even though NDVI is usually assumed to be based on red and near infrared calculations, because red edge NDVI is also utilized in these experiments. Red NDVI readings are an indication of the coverage of soil by leaves. Once leaves completely cover the row, as viewed from above the leaf canopy, differences in red NDVI readings fall into a narrow range, commonly from 0.85 to 1.0. Sensing differences in yield potential from leaf canopy closure on is difficult due to the narrow range of possible readings, referred to as “saturation” [24,25].

The plant pigments that are most involved in the photosynthetic process are chlorophylls a and b. These pigments absorb light in red and blue spectra and reflect green. Light reflectance from plants depends upon several factors such leaf surface area, surface properties, plant stress levels and internal structure [26]. Red and infrared wavelengths have been used frequently to assess the plant biomass [27]. There is more reflectance from plants in the near infrared (700–1400 nm) spectrum of light [28,29,30]. The Normalized Difference Vegetation Index (NDVI) is the most used vegetative index by researchers and practitioners for plant biomass prediction [31,32,33]. Measurement of normalized difference vegetative index (NDVI) and its conversion to in-season estimation of yield (INSEY) provides a convenient approach that can be used for variable in-season nitrogen (N) application [19,20,21,24,34,35] in corn. Within the red wavelength, green leaves have a reflectance of 20% or less in the 500 to 700 nm range (green to red) whereas within the red edge and near infrared wavelength, leaves reflect as much as 60% in the 700 to 1300 nm range (near infrared) [15]. In addition, the red spectrum is highly sensitive to low chlorophyll content (3–5 μg∙cm^−2^) whereas the red edge spectrum (700–750 nm) is sensitive to a wider range of chlorophyll (0.3–45 μg∙m^−2^) [15]. In pepper (*Capsicum annuum*), red edge was found to be the best wavelength describing the chlorophyll content [36].

Although the relationship between the red edge and leaf chlorophyll has been previously described [37,38], the application of the relationship on N status or yield prediction is unclear [36]. Part of the reason for uncertainty regarding the use of red edge NDVI is inconsistent past performance. For example, no correlation between red edge and leaf chlorophyll content in sugar beet (*Beta vulgaris*) was found by Demetriades-Shah and Steven [39]. In contrast, strong sensitivity of red edge NDVI to chlorophyll concentration in splash pine (*Pinus elliottii*) was reported in separate studies [40,41]. The performance of the red edge wavelength as a predictive tool for estimating potential yield depends not only on its sensitivity to chlorophyll concentration but also plant scattering properties inherent in total biomass, which change the peak light reflectance wavelength and the magnitude of reflectance from plants [38,41]. A strong relationship of red edge NDVI with yield was found at V12 stage in corn in recent North Dakota studies [25].

Plant growth stage is responsible for changes in the scattering behavior of light in several crops, including durum wheat (*Triticum durum*) [42], forage grasses [43], and barley (*Hordeum vulgare*, L.) [44]. In barley, the ratio of infrared to red changes with time, indicating that the ratio is growth dependent [44]. The reason of light scattering changes is the change in internal properties of plant leaves. Air space in mesophyll cells was reported to be less during younger growth stages compared to older leaves, which causes a change in the value of spectral reflectance [45]. Environmental stress such as water stress may also change reflective characteristics in red edge NDVI because water stress affects the internal structure of plant cells and leaves, which could influence the scattering of light [46].

The differences between red NDVI and red edge NDVI and their use as predictive tools have been studied in wheat [47], where it was found that red wavelength was more correlated to plant biomass. Numerous researchers have reported the differences between red NDVI and red edge NDVI in corn [20,48,49,50,51,52,53,54,55]. Generally, this research has found a weaker relationship with red NDVI and corn yield compared to earlier research with wheat. However, most of these studies did not include red edge NDVI. The red edge NDVI more closely relates to chlorophyll content than red NDVI [38]. The objective of this study was to determine the performance of red edge NDVI in yield prediction in corn compared to the yield prediction of red NDVI at early (V6) and later (V12) growth stages. The study also investigates the relative performance of two different red edge wavelengths through the use of two different ground-based active-optical sensors. The results of these experiments will be utilized to develop algorithms for the use of corn yield prediction with active-optical sensors for use in directing in-season N rates for corn.

## 2. Materials and Methods

### 2.1. Research Locations

Fifteen sites on farmer cooperator fields were used for N rate trials on corn in eastern and western North Dakota in 2013 (Table 1 and Table 2). The corn hybrid planted at each site was selected by the farmer and the site was planted at the same time as the rest of the field. The growers applied herbicides at their discretion but hand weeding was also carried where required. The experimental design was a randomized complete block with four replications and six N treatments; 0, 45, 90, 134, 179 and 224 kg∙N∙ha^−1^ as ammonium nitrate, hand broadcast about a week before planting. Each experimental unit (plot) was 6.1-m long and 3.05-m wide. Locations were categorized into high clay conventional-till sites and medium-textured conventional-till sites, eastern long-term no-till (continuous no-till system for at least 6 consecutive years) and western long-term no-till.

**Table 1:** GPS coordinates and soil series for field experiments in 2013.

Location	GPS Coordinates	Soil Type ^†^
Casselton	46°52′41.973′′ N, 97°14′55.894′′ W	Fine-silty, mixed, superactive, frigid Typic Endoaquolls
Durbin	46°51′22.072′′ N, 97°09′28.366′′ W	Fine, smectitic, frigid Typic Epiaquerts
Barney	46°15′07.560′′ N, 96°59′28.627′′ W	Coarse-loamy, mixed, superactive, frigid Aquic Pachic Hapludolls
Dwight	46°18′39.335′′ N, 96°47′12.237′′ W	Fine, smectitic, frigid Vertic Argialbolls
Gardner	47°10′28.482′′ N, 96°54′02.138′′ W	Fine, smectitic, frigid Typic Epiaquerts
Leonard-North	46°52′57.807′′ N, 97°17′44.945′′ W	Fine, smectitic, frigid Typic Epiaquerts
Walcott	46°30′02.359′′ N, 97°02′39.660′′ W	Coarse-loamy, mixed, superactive, frigid Aeric Calciaquolls
Leonard West	46°39′10.750′′ N, 97°18′12.980′′ W	Coarse-loamy, mixed, superactive, frigid Pachic Hapludolls
Arthur	47°06′50.963′′ N, 97°57′55.219′′ W	Coarse-silty, mixed, superactive, frigid Pachic Hapludolls
Rutland	45°57′50.176′′ N, 97°31′44.205′′ W	Fine, smectitic, frigid Pachic Vertic Argiudolls
Jamestown	46°45′58.571′′ N, 98°47′55.930′′ W	Fine-loamy, mixed, superactive, frigid Calcic Hapludolls
Mott	46°56′43.583′′ N, −102°19′10.919′′ W	Fine-loamy, mixed, superactive, frigid Typic Haplustolls
Richardton	46°35′0.095′′ N, −102°21′41.364′′ W	Fine-loamy, mixed, superactive, frigid Typic Haplustolls
Beach	46°49′3.0354′′ N, −103°59′40.451′′ W	Fine-silty, mixed, superactive, frigid Typic Haplustolls
New Leipzig	46°26′44.051′′ N, −101°56′31.379′′ W	Fine, smectitic, frigid Vertic Natrustolls

**^†^** Information collected from Soil Survey Staff, 2013.

**Table 2:** Tillage system, planting date and date of the first and second sensing of each location in 2013.

Locations	Soil/Tillage Category	Planting Date	V6	V6 GDD ^†^	V12	V12 GDD ^†^	Corn Variety
Casselton	High clay conventional-till	15/05/13	25/06/13	623	15/07/13	1095	P8640
Durbin	High clay conventional-till	15/05/13	25/06/13	623	15/07/13	1095	NA
Barney	High clay conventional-till	09/05/13	26/06/13	658	18/07/13	1227	P9917
Dwight	High clay conventional-till	16/05/13	26/06/13	605	09/07/13	898	DK 4837
Gardner	High clay conventional-till	10/05/13	25/06/13	581	10/07/13	1044	NutTech3A183
Leonard-North	High clay conventional-till	28/05/13	25/06/13	438	10/07/13	778	76R92
Walcott	Medium-textured conventional-till	18/05/13	02/07/13	700	18/07/13	1076	DeKalb 39-04
Leonard West	Medium-textured conventional-till	10/05/13	02/07/13	651	12/07/13	1107	76R92
Arthur	Medium-textured conventional-till	15/05/13	20/06/13	516	10/07/13	979	Mycogen-2T222
Rutland	Eastern no-till	08/05/13	18/0613	524	09/07/13	1012	Mycogen 2G-161
Jamestown	Eastern no-till	11/05/13	18/0613	424	09/07/13	870	Croplan 229VT2RTB
Mott	Western no-till	19/05/13	01/07/13	593	17/07/13	911	P8107
Richardton	Western no-till	13/05/13	No sensing	-	17/07/13	517	P8107
Beach	Western no-till	15/05/13	01/07/13	498	17/07/13	804	Pioneer D-97
New Leipzig	Western clay no-till	07/05/13	01/07/13	662	17/07/13	980	P8954XR

**^†^** GDD is growing degree days from planting to the date sensing at V6 or V12.

### 2.2. Soil Sampling and Analysis

Five soil sample cores were taken from each site before planting and treatment application using a 2.5-cm diameter hand probe to a depth of 0–15 cm for phosphorous (P), potassium (K), zinc (Zn), pH, and organic matter analysis, and three soil cores from 0 to 60 cm were obtained for determination of residual nitrate-N (Table 3). If N in the field was applied as a blend by the farmer to the rest of the field, P and K were applied by the researchers so that farmer application did not confound the N rate trial. When fertilizer P and K needed to be applied by the researchers, the sources were mono ammonium phosphate (11-52-0) and potassium chloride (0-0-60), at rates consistent with soil test analysis and NDSU Extension guidelines [56]. If Zn was deficient, zinc sulfate (36% granules) at a rate of 11 kg∙ha^−1^ Zn was applied using a spinner spreader as a broadcast at the time of N treatment application. If any site was suspected to be S deficient due to sandy texture and high rainfall or snowmelt, 112 kg∙ha^−1^ of calcium sulfate (0-0-0-20S) was applied at the time of N application. When unanticipated S deficiency appeared later in the season due to higher rainfall, an application of calcium sulfate at 22 kg∙ha^−1^∙S (112 kg∙ha^−1^ gypsum) was applied as granules over the top of the corn. Soil pH was analyzed using a 1:1 soil: deionized H_2_O solution method [57]; P was determined by the Olsen method [58], K was assessed using the 1-N ammonium acetate method [59]. The DTPA extraction method [60] coupled with atomic absorption spectroscopy detection was used for determination of available Zn. Organic matter was measured using the loss following ignition method [61].

**Table 3:** Relevant soil analysis for experimental locations.

Location	Depth	Nitrate	P	K	Zn	Organic Matter	pH
	cm	kg∙ha^−1^	---------mg kg^−1^---------			%	
**Casselton**	0–15	21	7	370	0.37	5.4	7.6
	0–61	55					
**Leonard North**	0–15	6	18	380	0.95	5.7	6.6
	0–61	15					
**Durbin**	0–15	6	34	460	0.62	5.9	7.5
	0–61	45					
**Arthur**	0–15	6	9	110	1.16	2.2	6.6
	0–61	12					
**Leonard West**	0–15	7	8	125	3.75	2.2	7.3
	0–61	12					
**Barney**	0–15	24	12	110	1.21	2.9	7.8
	0–61	91					
**Walcott**	0–15	13	9	347	0.78	4.2	6.9
	0–61	29					
**Dwight**	0–15	35	63	540	2.37	4	7.7
	0–61	26					
**Rutland**	0–15	20	8	415	0.72	6.1	7
	0–61	54					
**Jamestown**	0–15	10	8	220	1.14	3.3	5.7
	0–61	12					
**Dwight**	0–15	12	8	185	0.45	3.5	7.9
	0–61	72					
**Mott**	0–15	18	4	230	0.95	5.2	7.6
	0–61	10					
**Richardton**	0–15	11	33	170	0.65	3.2	5.1
	0–61	9					
**Beach**	0–15	17	22	300	0.85	3	6.2
	0–61	7					
**New Leipzig**	0–15	23	16	560	1.46	5.2	5.6
	0–61	18					

### 2.3. Sensor Description and Sensing Procedure

The ground-based active-optical (GBAO) sensors use diodes to generate modular light pulses of particular wavelengths absorbed by plant tissues. Two handheld GBAO sensors were used for this study: GreenSeeker™ (GS; Trimble Navigation Limited, Sunnyvale, CA, USA) and the Holland Scientific Crop Circle™ A470 sensor (CC; Holland Scientific, Inc., Lincoln, NE, USA). The GS sensor measures incident and reflected light from plants at 660 ± 15 nm (red) and 770 ± 15 nm (NIR). In the GS, light is emitted from diodes in alternating bursts of different duration such that the visible source pulses for 1 ms and then the NIR diode source pulses for 1 ms at 40,000 Hz. Each burst from a given source amounts to about 40 pulses before pausing for the other diode to emit its radiation (another 40 pulses). The illuminated area is about 60 cm wide by 1 cm long, with the long dimension positioned perpendicular to the direction of travel. The field of view is approximately constant for heights between 60 and 120 cm above the canopy because of light collimation within the sensor. Outputs from the sensor are red NDVI and simple ratio (red/NIR).

The CC sensor simultaneously emits three bands; two in the visible range (red 650 nm, red edge 730 nm) and one in the NIR (760 nm). The light source of the CC is a modulated polychromatic LED array. It can emit and measure light spectrums in the range from 430 nm to 850 nm band width (BW). The sensor has a measurement filter range including wavelengths of 450 nm (BW ± 20 nm), 550 nm (BW ± 20 nm), 650 nm (BW ± 20 nm), 670 nm (BW ± 11 nm), 730 nm (BW ± 10 nm) and 760 nm (LWP).

The sensor was calibrated using software developed by Holland Scientific. Measurements can be collected at a rate of 2–20 readings per second, so each recorded value in a 20 foot length of plot, moving about 5 km∙hr^−1^ is the average of about 4000 readings. Outputs of the sensor are reflectance values that allow calculation of vegetation indices.

The formula for red NDVI and red edge NDVI follows:

(1) $$Red\;NDVI=\frac{NIR-Red}{NIR+Red}$$

(2) $$Red\;Edge\;NDVI=\frac{NIR-Red\;Edge}{NIR+Red\;Edge}$$

Wavelengths values used for GreenSeeker^®^ and Holland Scientific Crop Circle Sensor^®^ are defined below:

GS emits four bands: red (660 nm), two red edge (710 nm and 735 nm), and near infrared (774 nm):

NDVI = (NIR − Red)/(NIR + Red)

(774 nm reading − 660 nm reading)/(774 nm + 660 nm)

Or red edge NDVI (710 nm)

(774 nm reading − 710 nm reading)/(774 nm + 710 nm)

Or red edge (735 nm)

(774 nm reading − 735 nm reading)/(774 nm + 735 nm)

CC emits three bands: red (670 nm), red edge (730 nm), and near infrared (760 nm):

NDVI = (NIR − Red)/(NIR + Red)

(760 nm reading − 670 nm reading)/(760 nm + 670 nm)

Or red edge NDVI (730 nm)

NDVI= (NIR − Red Edge)/(NIR + Red Edge)

(760 nm reading − 730 nm reading)/(760nm + 730 nm)

The sensing with GS and CC were conducted at the V6 growth stage and V12 growth stage. Sensor readings were obtained 50 cm over the top of the corn whorls from the middle row of each plot. All reflectance data (NDVI) were inserted within the generalized expression:

NDVI = (NIR − red or red-edge)/(NIR + red or red-edge)
(3)

Within each experimental unit, 30–50 individual readings were obtained. The mean of GS and CC readings were calculated using in-house macro programs for Visual Basic within Excel [62]. To normalize NDVI over a range of sites where small differences in growth stage were found (*i.e.*, V5.5 to V6.5) the normalizing factor of INSEY was used. The INSEY (in-season estimate of yield) [19] was calculated by dividing the NDVI with the growing degree days from planting date to date of sensing using the closest NDAWN data to each site [63]. Sensing was conducted by positioning the GS and CC at an approximate distance of 50 cm above the canopy, resulting in similar intensity of reflectance at each site and each growth stage reading [64].

### 2.4. Harvesting and Statistical Analysis

Harvest was conducted between 15 September and 15 October 2013. The sensed row of each experimental unit (plot) was hand harvested, leaving the outside ears at each end of the plot intact due to alleyway effect, taken from the field and then shelled using an Almaco^®^ corn sheller (Almaco, Nevada, IA, USA). Grain moisture and test weight was determined on a subsample of shelled grain using a Dickey-John GAC500XT moisture-test weight instrument (Dickey-John, Auburn, IL, USA).

Regression analyses were conducted on sensor readings converted to INSEY and yield with yield as the dependent variable and INSEY as the independent variable. The exponential regression model was superior to other possible regression models, so the regression relationships in Table 4 and Table 5 are expressed from the exponential model for red and red edge generated data. Multiple regression analysis was used to determine whether the data should be segregated into long-term no-till sites, high clay conventional and, medium texture conventional sites. The analysis confirmed that segregation of the data into those categories improved the relationship between INSEY and yield [25].

The coefficient of determination (r^2^) value was used to evaluate the relationship of crop yield and sensor reading at V6 and V12. The SAS program for Windows V9.2 (SAS Institute, Cary, NC, USA), using the procedure PROC REG, was used to calculate the r^2^ and evaluate regression models of. SAS procedure GLM was used to compare the N treatments for treatment differences in yield and the INSEY calculated from five sensor wavelengths. A *p*-value of 5% probability was used to differentiate between treatment differences using the LSD derived from ANOVA. Coefficient of variation (CV) was also calculated from wavelength INSEY, and bar graphs were prepared to check the variation of red and red edge wavelength INSEY at V6 and V12. In addition, bar and line graphs between treatment and INSEY as well as wavelength and INSEY were also prepared to check the sensitivity and response of wavelength at different treatment levels as well to compare the wavelength peaks within their category.

**Table 4:** Exponential regression equations for Yield and INSEY for five wavelengths of two ground-based active-optical sensors under high clay (*n* = 143) and medium textured conventional-till (*n* = 71) sites at V6 and V12 corn growth stages.

Corn Growth Stage	Sensor/Wavelength	Regression Equation and r^2^ High Clay Soils	Sig ^†^	Regression Equation and r^2^ Medium Textured Soils	Sig ^†^
V6	CC^††^/730 nm (red edge)	y = 6709.78e^1052.3x^ r^2^ = 0.20	*	y = 6351.09e^2807x^ r^2^ = 0.40	***
	CC/670 nm (red)	y = 6915.99e^427.26x^ r^2^ = 0.17	*	y = 5787.54e^1503.4x^ r^2^ = 0.42	***
	GS/660 nm (red)	y = 7816.47e^267.19x^ r^2^ = 0.06	NS	y = 5312.75e^1452.4x^ r^2^ = 0.39	**
	GS/710 nm (red edge)	y = 7722.99e^600.1x^ r^2^ = 0.07	NS	y = 5893.79e^2693.3x^ r^2^ = 0.41	***
	GS/735 nm (red edge)	y = 7437.18e^2398.2x^ r^2^ = 0.08	NS	y = 5747.19e^9351.6x^ r^2^ = 0.33	**
V12	CC/730 nm (red edge)	y = 4492.97e^2002.9x^ r^2^ = 0.18	*	y = 2111.65e^4626.1x^ r^2^ = 0.58	***
	CC/670 nm (red)	y = 3736.41e^1273.9x^ r^2^ = 0.18	*	y = 1423.68e^2914.9x^ r^2^ = 0.47	***
	GS/660 nm (red)	y = 3650.33e^1324.7x^ r^2^ = 0.20	*	y = 995.97e^3431.9x^ r^2^ = 0.47	***
	GS/710 nm (red edge)	y = 6139.93e^999.23x^ r^2^ = 0.06	NS	y = 1511.98e^49^°^5.3x^ r^2^ = 0.55	***
	GS/735 nm (red edge)	y = 7078.74e^2065.4x^ r^2^ = 0.04	NS	y = 3583.75e^8914.5x^ r^2^ = 0.49	***

^†^ NS denotes non-significance at *p* < 0.05; *** denotes significance at 0.001; ** denotes significance at 0.01; * denotes significance at 0.05; ^††^ CC is Holland Crop Circle™ and GS is GreenSeeker™.

**Table 5:** Exponential regression equations of Yield and INSEY from five wavelengths of two ground-based active-optical sensors in eastern North Dakota (*n* = 47) and western North Dakota no-till (*n* = 95) systems at V6 and V12 growth stages of corn.

Corn Growth Stage	^††^ Sensor/Wavelength	Regression Equations and r^2^ for Eastern ND Long-Term No-till Sites	Sig ^†^	Regression Equations and r^2^ for Western ND Long-Term No-Till Sites	Sig ^†^
**V6**	**CC/730 nm (red edge)**	y = 7525.95e^456.92x^ r^2^ = 0.00	NS	y = 4464.06e^3529.3x^ r^2^ = 0.32	**
	**CC/670 nm (red)**	y = 10096.92e^−604.5x^ r^2^ = 0.01	NS	y = 3700.77e^2211.2x^ r^2^ = 0.38	***
	**GS/660 nm (red)**	y = 15252.57e^−1310x^ r^2^ = 0.06	NS	y = 3331.57e^1775.7x^ r^2^ = 0.39	**
	**GS/710 nm (red edge)**	y = 10362.54e^−1101x^ r^2^ = 0.02	NS	y = 3805e^3245.8x^ r^2^ = 0.37	**
	**GS/735 nm (red edge)**	y = 9581.11e^−2145x^ r^2^ = 0.01	NS	y = 3687.96e^10657x^ r^2^ = 0.36	**
**V12**	**CC/730 nm (red edge)**	y = 794.96e^5744.7x^ r^2^ = 0.39	*	y = 4564.26e^1201.1x^ r^2^ = 0.34	**
	**CC/670 nm (red)**	y = 2474.80e^1555.3x^ r^2^ = 0.06	NS	y = 4479.52e^650.89x^ r^2^ = 0.29	*
	**GS/660 nm (red)**	y = 3591.15e^1088.3x^ r^2^ = 0.02	NS	y = 5005.42e^434.32x^ r^2^ = 0.20	*
	**GS/710 nm (red edge)**	y = 1663.77e^3531.2x^ r^2^ = 0.18	*	y = 5079.39e^759.99x^ r^2^ = 0.23	*
	**GS/735 nm (red edge)**	y = 1733.03e^10841x^ r^2^ = 0.36	*	y = 5262.99e^2122.4x^ r^2^ = 0.19	*

**^†^** NS denotes non-significance at *p* < 0.05; * denotes significance at 0.05; ** denotes significance at 0.01; *** denotes significance at 0.001; ^††^ CC is Holland Crop Circle™ and GS is GreenSeeker™.

## 3. Results

### 3.1. Regression Analysis

The exponential relationships between INSEY and yield at V6 and V12 stages of corn where five wavelengths were used to compare the coefficient of determination (r^2^) between corn yield and sensor reading are provided in Table 4. The exponential relationships were roughly linear, but the r^2^ values from the exponential models were nearly always superior to that of the linear model. In high clay sites at V6 using the CC, the red edge NDVI-based INSEY was significantly related to yield whereas both GS red edge NDVI-based INSEY was unrelated to yield. At V12 CC red NDVI, CC red edge NDVI, and GS red NDVI based INSEY were related to yield, while both wavelengths of GS red edge NDVI were not. In medium textured sites, the INSEY derived from wavelengths of both active-sensors at both growth stages were significantly related to yield (Table 4).

In eastern North Dakota (ND) long-term no-till sites, there were no INSEY relationships between yield and sensor readings at V6 (Table 5). In western ND long-term no-till sites, there were significant relationships between INSEY and yield using both sensors and all wavelengths at V6 (Table 5). In previous research, there was no clear trend in the effect of crop residues on wavelength absorption and reflectance compared to soil; residue coverage of the soil can vary, with the effect of residue coverage influenced by residue moisture content and the origin of residue [65,66,67]. More consistent identification and quantification of residue appears to be only possible using bands in wavelengths greater than 1110 nm. It is noteworthy that eastern no-till sites tended to have denser residue cover, partially due to the tendency of eastern site cooperators to utilize more fall cover crops, and the tendency at some eastern sites to follow small grain with corn, whereas western sites tended to follow a lower residue crop. At V12 in western ND there were no INSEY relationships with yield in eastern ND sites using red NDVI or red edge NDVI. Using red edge NDVI, the relationship between INSEY and yield was significant at eastern ND long-term no-till sites. At V12 in eastern ND, the relationships between INSEY and yield were significant with both sensors and all wavelengths. The r^2^ values in eastern ND for V12 tended to be less than those at V6.

### 3.2. Wavelength Sensitivity and Absorption Spectra

**Figure 1:**
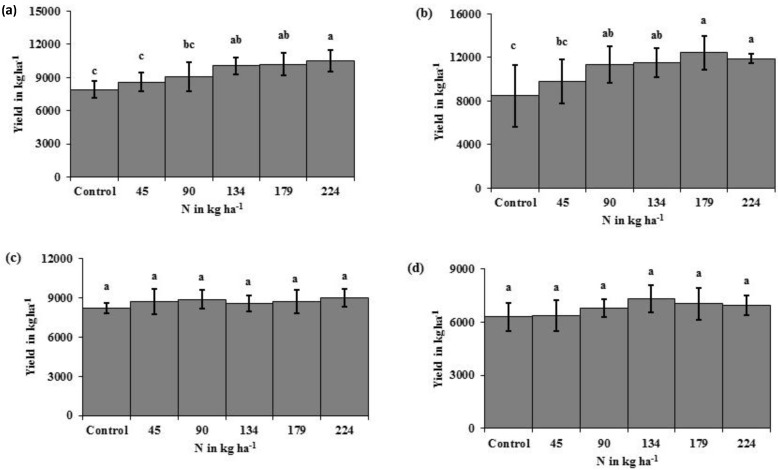
Corn yield difference in response to N fertilizer rates under high clay conventional-till (**a**); medium textured conventional-till (**b**); eastern ND no-till (**c**); and western ND no-till (**d**) sites. Bars with different letters indicate significance at *p* < 0.05. Bars represent the range of yields within each N rate.

**Figure 2:**
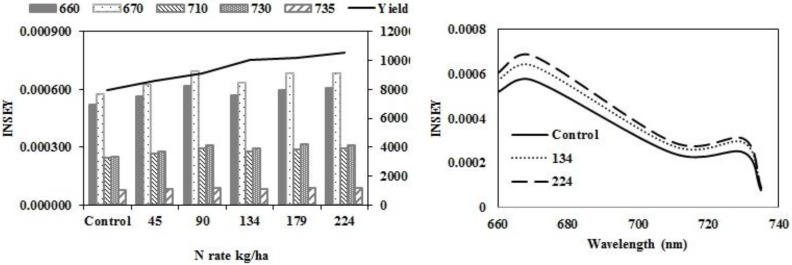

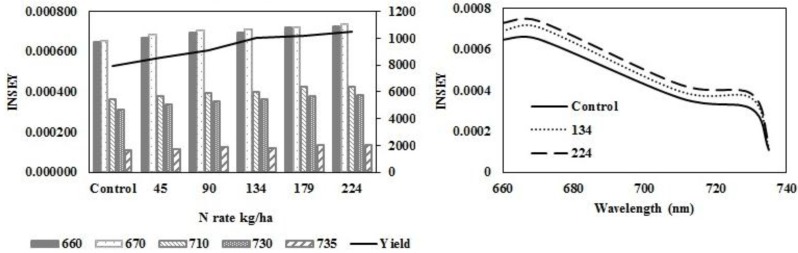
Variation of INSEY for corn at high clay conventional-till sites calculated from CC active-sensor spectra (670 nm and 730 nm) and GS spectra (660 nm, 710 nm and 735 nm) with N rate compared to yield variation with N rate at V6 **(upper left)** and V12 **(lower left)**. Trend for INSEY at three N rates (control, 134∙kg∙ha^−1^ and 224∙kg∙ha^−1^) over the five wavelengths represented as a continuous spectrum is displayed at V6 in figures at (**upper right)** and at V12 in **(lower right)**.

The N rate treatments resulted in significant yield differences, with increased yield as N rate increased in eastern ND high clay conventional-till sites (Figure 1a) and eastern ND medium textured conventional-till sites (Figure 1b). There were no yield responses due to N rate at eastern ND no-till sites (Figure 1c) or western ND no-till sites (Figure 1d). The INSEY varied similarly to yield. In Figure 2a,c, and Table 6, with yield and INSEY at eastern ND high clay sites, yield response and INSEY response to N rate were similar. The sensitivity, which is the ability of a measurement to relate to an outcome, was greater for the INSEY developed from red wavelengths of the GS (660) and CC (670) compared to red edge INSEYs (Figure 2b,d, Table 6), meaning that the separation of INSEY values with N rate was greater for INSEY developed under the red wavelengths than those developed under red-edge. At V6, although there appears to be a similar response of 710 nm and 730 nm (Figure 2a) wavelengths with N rates the INSEY relationship using the 710 nm red edge NDVI was not significant, while the red edge INSEY relationship using 730 nm was significant (Table 4). At V12, the sensitivity of both the 710 nm and 730 nm derived INSEY was similar (Figure 2b) and the relationship to yield was also similar (Table 4 and Table 6). The INSEY values at high clay sites increased from V6 to V12, which is commonly observed due to greater biomass with growth (Figure 2a,b). The absorption spectrum trends of INSEY with wavelength at three different N rates were similar at V6 and V12 (Figure 2b,d).

**Table 6:** Treatment differences between INSEY developed from five wavelengths utilizing two ground-based active-optical sensors in eastern high clay (*n* = 143) and eastern medium-textured (*n* = 71) categories under conventional tillage at two corn growth stages from nitrogen rate treatments.

Soil Type/Tillage System	Growth Stage	N Treatment kg·N·ha^−1^	INSEY ^†^ X 10,000				
			Holland Crop Circle		GreenSeeker		
			Red Edge 730 *	Red 670	Red 660	Red Edge 710	Red Edge 735
High Clay	V6	0	0.000249 b	0.000572 a	0.000519 a	0.000244 a	0.000078 a
		45	0.000275 ab	0.000623 a	0.000564 a	0.000265 a	0.000082 a
		90	0.000311 ab	0.000694 a	0.000618 a	0.000296 a	0.000088 a
		135	0.000293 ab	0.000636 a	0.000571 a	0.000277 a	0.000085 a
		179	0.000315 a	0.000685 a	0.000598 a	0.000291 a	0.000089 a
		224	0.000310 ab	0.000682 a	0.000605 a	0.000295 a	0.000090 a
	V12	0	0.000311 d	0.000650 c	0.000647 b	0.000362 b	0.000110 c
		45	0.000338 cd	0.000684 bc	0.000668 b	0.000378 b	0.000114 c
		90	0.000350 bc	0.000703 ab	0.000694 ab	0.000393 ab	0.000122 bc
		135	0.000365 abc	0.000711 ab	0.000693 ab	0.000397 ab	0.000120 c
		179	0.000376 ab	0.000720 ab	0.000720 a	0.000423 a	0.000135 ab
		224	0.000382 a	0.000737 a	0.000727 a	0.000426 a	0.000137 a
Medium- textured	V6	0	0.000160 a	0.000360 a	0.000423 a	0.000190 a	0.000062 a
		45	0.000164 a	0.000365 a	0.000452 a	0.000206 a	0.000063 a
		90	0.000190 a	0.000426 a	0.000502 a	0.000225 a	0.000065 a
		135	0.000186 a	0.000399 a	0.000483 a	0.000223 a	0.000066 a
		179	0.000212 a	0.000460 a	0.000515 a	0.000242 a	0.000072 a
		224	0.000196 a	0.000429 a	0.000503 a	0.000235 a	0.000069 a
	V12	0	0.000306 c	0.000646 b	0.000651 b	0.000359 c	0.000104 c
		45	0.000337 bc	0.000675 ab	0.00068 ab	0.000388 bc	0.000114 bc
		90	0.000357 ab	0.000702 a	0.000706 a	0.000406 ab	0.000126 ab
		135	0.000362 ab	0.000704 a	0.000696 ab	0.000403 ab	0.000125 ab
		179	0.000374 a	0.000713 a	0.000711 a	0.00042 a	0.000135 a
		224	0.000365 ab	0.000704 a	0.000701 a	0.000414 ab	0.00013 a

^†^ INSEY is the sensor reading divided by growing degree days from planting date; * Wavelength in nm.

**Figure 3:**
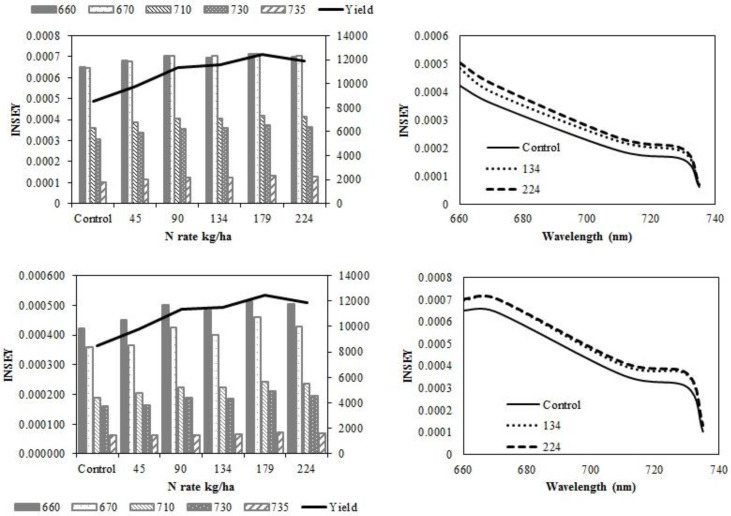
Variation of INSEY for corn at medium-textured conventional-till sites calculated from CC active-sensor spectra (670 nm and 730 nm) and GS spectra (660 nm, 710 nm and 735 nm) with N rate compared to yield variation with N rate at V6 **(upper left)** and V12 **(lower left)**. Trend for INSEY at three N rates (control, 134 kg∙ha^−1^ and 224 kg∙ha^−1^) over the five wavelengths represented as a continuous spectrum is displayed at V6 **(upper right)** and at V12 **(lower right)**.

In eastern ND medium textured sites (Figure 3a–d), wavelength sensitivity of active-optical sensor INSEY to yield was similar at V6 (Figure 3a) and V12 (Figure 3c) with sensitivity greater with red NDVI derived INSEY ranges at V6 and V12. At V6 and V12, the sensitivity of red NDVI INSEY in both sensors to N rates was similar and both were similarly related to yield at both growth stages (Table 4). The trend for greater INSEY with N rate was less clear at V6 (Table 6). At V6, there were no significant relationships between INSEY and yield for all wavelengths. At V12, the relationships between INSEY and yield were significant for all wavelengths. Yield is influenced by factors other than N rate [68,69,70,71], which is why an N non-limiting area should be established within a field where active-optical sensors will be used to help determine in-season supplemental N fertilization [72].

**Figure 4:**
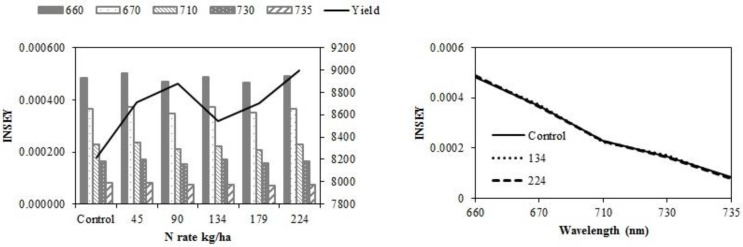

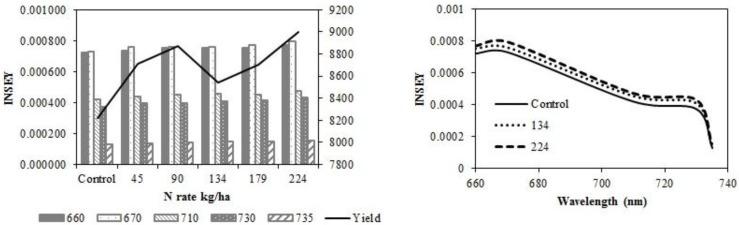
Variation of INSEY for corn at eastern no-till sites calculated from CC active-sensor spectra (670 and 730 nm) and GS spectra (660, 710 and 735 nm) with N rate compared to yield variation with N rate at V6 **(upper left)** and V12 **(lower left)**. Trend for INSEY at three N rates (control, 134 kg·ha^−1^ and 224 kg·ha^−1^) over the five wavelengths represented as a continuous spectrum is displayed at V6 **(upper right)** and at V12 **(lower right)**.

In eastern ND no-till sites, yield did not increase with N rate. At V6, there was no relationship between INSEY and yield (Table 5) or INSEY or N rate (Table 7). Yield trends depicted in Figure 4a,c are not significant. At V12, the r^2^ between CC 730 nm NDVI derived INSEY and yield and the GS 735 nm NDVI derived INSEY and yield were significant. The INSEY from all wavelengths of both sensors at V12 was also related to N rate (Table 7).

**Table 7:** Treatment differences between INSEY developed from five wavelengths utilizing two ground-based active-optical sensors in eastern North Dakota (ND) (*n* = 47) and western ND no-till (*n* = 95) systems at two corn growth stages from nitrogen rate treatments.

Soil Category	Growth Stage	N Treatment kg·N·ha^−1^	INSEY ^†^ X 10,000				
			Holland Crop Circle		GreenSeeker		
			Red Edge 730	Red 670	Red 660	Red Edge 710	Red Edge 735
**Eastern ND**	**V6**	0	1.65 a	3.65 a	4.83 a	2.28 a	0.83 a
		45	1.72 a	3.73 a	5.03 a	2.35 a	0.83 a
		90	1.52 a	3.46 a	4.67 a	2.12 a	0.76 a
		135	1.70 a	3.72 a	4.85 a	2.23 a	0.76 a
		179	1.58 a	3.52a	4.65 a	2.09 a	0.73 a
		224	1.65 a	3.66 a	4.89 a	2.28 a	0.76 a
	**V12**	0	3.74 c	7.31 b	7.22 b	4.21 b	1.29 c
		45	3.97 bc	7.57 ab	7.38 ab	4.37 ab	1.36 bc
		90	3.99 bc	7.61 ab	7.55 ab	4.52 ab	1.42 abc
		135	4.09 ab	7.60 ab	7.52 ab	4.54 ab	1.47 ab
		179	4.16 ab	7.74 ab	7.55 ab	4.54 ab	1.46 abc
		224	4.31 a	7.96 a	7.70 a	4.73 a	1.54 a
**Western ND**	**V6**	0	9.45 a	2.35 a	3.53 a	1.51 a	4.88 a
		45	9.07 a	2.26 a	3.33 a	1.41 a	4.74 a
		90	1.15 a	2.69 a	3.85 a	1.68 a	5.40 a
		135	1.09 a	2.56 a	3.92 a	1.74 a	5.68 a
		179	1.14 a	2.73 a	3.98 a	1.79 a	5.63 a
		224	1.08 a	2.58 a	3.83 a	1.69 a	5.40 a
	**V12**	0	2.85 a	5.61 a	6.27 a	3.30 a	1.01 a
		45	2.85 a	5.58 a	6.12 a	3.26 a	1.00a
		90	3.33 a	6.48 a	6.70 a	3.64 a	1.12 a
		135	3.19 a	6.16 a	6.56 a	3.61 a	1.14 a
		179	3.36 a	6.45 a	6.87 a	3.80 a	1.20 a
		224	3.10 a	5.92 a	6.40 a	3.47 a	1.08 a

**^†^** INSEY is the sensor reading divided by growing degree days from planting date.

**Figure 5:**
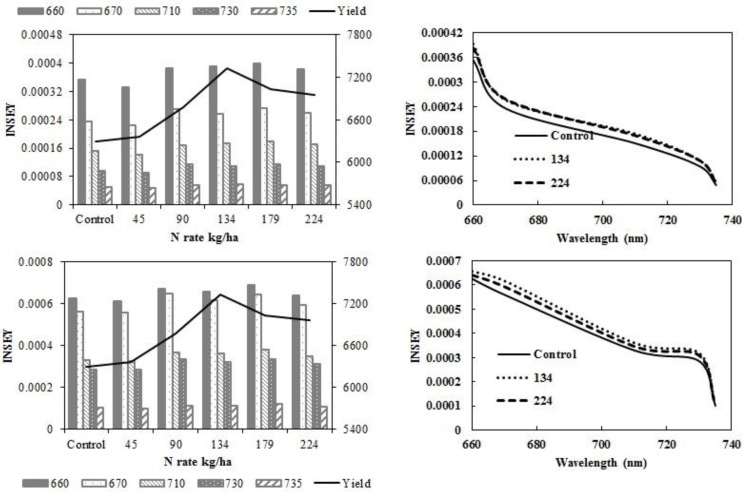
Variation of INSEY for corn at western ND no-till sites calculated from CC active-sensor spectra (670 and 730 nm) and GS spectra (660, 710 and 735 nm) with N rate compared to yield variation with N rate at V6 **(upper left)** and V12 **(lower left)**. Trend for INSEY at three N rates (control, 134 kg·ha^−1^ and 224 kg·ha^−1^) over the five wavelengths represented as a continuous spectrum is displayed at V6 **(upper right)** and at V12 **(lower right)**.

At western ND no-till sites, there was no response to N. However, there were significant relationships between all wavelengths of both sensors and yield (Table 4), and N rate at V12 (Table 7). The sensitivity of INSEY to yield was low at V6 and V12 in western ND no-till sites. The lack of relationship was particularly evident at the lower N rates (Figure 5).

### 3.3. Coefficient of Variation among Treatments

**Figure 6:**
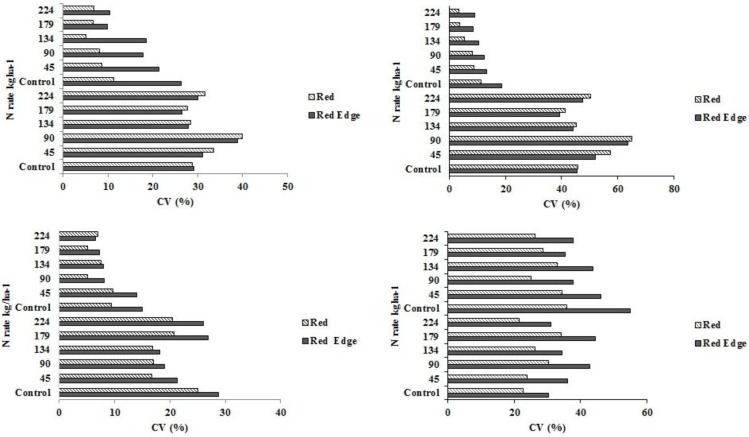
Percent CV (coefficient of variation) calculated from INSEY derived from sensor red or red edge NDVI in response to N rate under high clay conventional-till (**upper left**); medium textured conventional-till (**upper right**); eastern ND no-till (**lower left**); and western ND no-till (**lower right**) sites at V6 and V12 corn growth stages.

During early growth stages, the leaf surface area obtained from NADIR-aimed imagery is small, therefore most sensor reflectance comes from the soil surface. Variations in residue cover and type as well as other surface soil and early plant size variation due to uneven emergence probably contributed to the high coefficient of variation (CV) values with red NDVI- and red edge NDVI-based INSEY under all soil and tillage system categories (Figure 6a–d). The CVs at V12 were generally less than those at V6 for all categories except at western no-till sites. The CVs for the control (zero-N) treatments were larger than other treatments. The CVs were particularly large in western no-till control plots, which may be one reason why there was no statistical response to N at these sites. In previous research, the N response in long-term no-till fields was low, presumably due to greater efficiency of N utilization in long-term no-till systems in North Dakota [70]. Future work will explore methods to filter out residue effects from crop growth crop canopy NDVI measurements.

## 4. Discussion

It was necessary to segregate different soils and tillage categories to help explain relational differences in this study [25]. At eastern ND high clay sites, the red NDVI- and red edge NDVI- based INSEY were similar in their prediction of yield at V6 and V12. However, although red or red edge sensors could be used to estimate corn yield at V6 or V12, there are some fundamental differences in their modes of action. Red-based sensors are useful at early (V6) growth stages due to their reliance on the proportion of soil covered with leaves for measurement differences. The red edge-based sensors do not respond to leaf area differences, and measure differences in leaf color, “tint”, which is highly related to chlorophyll content. Therefore, we found that the red edge NDVI-based INSEY was predictive at V6 and V12 stages consistently at our sites. Similar results were being reported where red- and red edge-based sensors were compared at V6 [71]. Algorithms for use in directing in-season N application for corn have been developed [34] using two different wavelength ranges from two different sensors (GreenSeeker and Crop Circle ACS-210). Several N rate algorithms using red and red edge wavelengths in corn have been developed [69]; however, algorithms are only available for sensor wavelength and growth stage that have yield predictive capabilities.

The weakness of the red wavelength to estimate yield at V12 stage was due to the saturation effect. Several studies have suggested early (V6) sensing and N application in corn [34,71,72,73] to alleviate N deficiencies; however, the relationship between yield and INSEY tends to increase when the crop is sensed at later growing stages, especially using red edge NDVI-based INSEY [71]. In addition, N status of a crop is not only related to leaves, but N content of stems as well [74]. A “vertical footprint” of sensor measurements was used to characterize N uptake in corn [75]. While this approach is not currently practical in commercial corn production, it emphasized that use of an over-canopy optical sensor has limitations for N status prediction simply because it is only using the upper leaf canopy for its assessment.

The problem associated with low yield predictability using the red NDVI-based INSEY once corn leaves cover the row, as at V12, is due to the difficulty in sensor ability to distinguish differences in plant health because reflection differences are small, even though to an observer clear differences in yellow to green tints are evident [76]. Similar results were reported [24] where low sensitivity of corn to the red wavelengths at V12 stage was recorded. Not only was a better relationship of red edge measurements to N rate found, but also a higher correlation of chlorophyll content with advances in growth stage.

Differences in the INSEY and yield relationships between high clay and medium texture soils were probably due to high rainfall in May and early June through V6. Rainfall was more than 10 cm above normal rainfall from planting through V6 [63]. In high clay soils, small pore saturation with water results in denitrification [77,78]. At the high clay sites, yields increased to the 224 kg∙ha^−1^ rate whereas medium textured sites seldom responded to N rates greater than 179 kg∙ha^−1^ although they obtained greater yields than high clay sites. The tendency for medium textured soils to drain better compared to high clay soils aided their greater N use efficiency, while apparently not leaching N excessively.

The relationships found in this study were specific to the wavelengths and the instrument used to generate the data. Crop Circle red NDVI-based INSEY regression equations were different than GS red NDVI-based INSEY relationships for example. This study is not the first conclude that relationships are defined by the instrument and wavelength used [78]. In addition, release of algorithms for yield prediction and use in directing in-season N application to any crop is not recommended with a one-year data base. Multiple years and environments would be necessary to recommend any active-optical sensor algorithm, and they must be specific to the instrument and wavelengths utilized in developing the algorithm. However, this study showed that the red NDVI-based or red edge NDVI-based INSEY data from either instrument could be used at V6 to develop a yield prediction-based in-season N rate algorithm for corn, and the red edge NDVI-based INSEY from the Crop Circle, or the GreenSeeker using the 710 nm wavelength, could be used to develop a yield prediction-based in-season N rate algorithm for corn at V12.

These results were generated from 15 sites across a geography of over 500 km in longitude and 150 km in latitude in North Dakota, spanning conventional tilled and long-term no-till sites and sites with high clay as well as those with lower clay content. Environmental conditions were different at each site with respect to rainfall and its distribution. Each site was seeded to a different hybrid. The yields within the study ranged from about 6000 kg∙ha^−1^ to about 12,000 kg∙ha^−1^, which spans the range of average yield of all major corn growing US states [79]. The diversity of conditions of these experiments and the similar response between sites of similar soil and tillage strongly suggest that these results would be expected to be replicated by anyone conducting a similar study on corn using these instruments.

## 5. Conclusions

Corn yield prediction using the GS red NDVI INSEY and CC red NDVI INSEY were similar. The red NDVI-based INSEY and red edge NDVI-based INSEY were similar in yield prediction at V6. At V12, the red edge NDVI-based INSEY provided consistently greater r^2^ values when predicting yield. Long-term no-till sites gave inconsistent INSEY measurements at V6, presumably due to ground residue cover. The r^2^ values of the relationships of INSEY and yield in all soil and tillage categories were superior at V12 compared to the V6 growth stage. Within the red edge spectrum of the GS sensor, the 710 nm wavelength was superior at corn yield prediction compared to 735 nm.
